# The neuroanatomy of autism – a developmental perspective

**DOI:** 10.1111/joa.12542

**Published:** 2016-09-12

**Authors:** Alex P. A. Donovan, M. Albert Basson

**Affiliations:** ^1^ Department of Craniofacial Development and Stem Cell Biology, and MRC Centre for Neurodevelopmental DisordersKing's College LondonLondonUK

**Keywords:** autism, cerebellum, development, dysplasia, frontal cortex, minicolumns, neuroanatomy, overgrowth, regionalisation

## Abstract

Autism Spectrum Disorders (ASDs) are a heterogeneous group of neurodevelopmental disorders that are diagnosed solely on the basis of behaviour. A large body of work has reported neuroanatomical differences between individuals with ASD and neurotypical controls. Despite the huge clinical and genetic heterogeneity that typifies autism, some of these anatomical features appear to be either present in most cases or so dramatically altered in some that their presence is now reasonably well replicated in a number of studies. One such finding is the tendency towards overgrowth of the frontal cortex during the early postnatal period. Although these reports have been focused primarily on the presumed pathological anatomy, they are providing us with important insights into normal brain anatomy and are stimulating new ideas and hypotheses about the normal trajectory of brain development and the function of specific anatomical brain structures. The use of model systems that include genetic model organisms such as the mouse and, more recently, human induced pluripotent stem cell‐derived brain organoids to model normal and pathological human cortical development, is proving particularly informative. Here we review some of the neuroanatomical alterations reported in autism, with a particular focus on well‐validated findings and recent advances in the field, and ask what these observations can tell us about normal and abnormal brain development.

## Introduction

ASD is characterised by persistent difficulties in communication and social interactions and restricted, repetitive patterns of behaviour, interests or activities. A number of neuroanatomical abnormalities have been reported to be associated with ASD. These include region‐specific volumetric changes, quantified by magnetic resonance imaging (MRI), specific alterations in grey or white matter, long‐range connectivity of different brain regions as inferred from diffusion tensor imaging (DTI) studies, and fine micro‐structural or cellular changes detected in postmortem brain tissue. These findings have provided important information on normal brain anatomy and have stimulated further research into the fundamental processes that shape brain development. By showing us intriguing examples of pathological anatomy, these studies will continue to reveal critical insights into the genetic, cellular and anatomical underpinnings of brain development.

In this manuscript, we will start by reviewing some of the most intriguing anatomical differences described between ASD and neurotypical brains and then move on to discuss what these observations might teach us about how specific aspects of brain development are regulated to ensure normal anatomy and function.

## Brain regions associated with the pathology of Autism Spectrum Disorders

### Cerebellum

The cerebellum, a highly foliated, neuron‐dense structure of the anterior‐dorsal hindbrain, is located inferior to the occipital lobe of the cerebral cortex and superior to the pons and brainstem. The role of the cerebellum in proprioception and fine motor control is well‐established but recent studies also strongly implicate the cerebellum in higher order cognitive functions such as language, cognitive processing and affective regulation (reviewed in Strick et al. 2009; Basson & Wingate, 2013; Becker & Stoodley, 2013).

Hypoplasia of the central cerebellar vermis lobules (VI+VII) was the first neuroanatomical change detected in the brains of ASD patients (Courchesne et al. 1988). Since then, many studies have reported cerebellar abnormalities in ASD (reviewed in Becker & Stoodley, 2013). A recent meta‐analysis conducted by Stoodley used anatomic likelihood estimate analysis on 17 voxel‐based morphometry studies of grey matter volume to compare the cerebella of male ASD patients with age‐matched neurotypical controls at various time points (Stoodley, 2014). This analysis found consistent volumetric decreases in the inferior cerebellar vermis (lobule XI), right crus I, and left lobule VIIIB. However, it is important to note that these specific findings are not observed consistently in all cohorts or studies. For example, some studies have found no changes in cerebellar volume (Piven et al. 1997), and the Courchesne group has reported individuals that also show the opposite phenotype, i.e. cerebellar hyperplasia (Courchesne et al. 1994).

The discrepancy between different studies is indicative of a recurring problem in the field. ASD is an extremely heterogeneous condition and patient selection is likely to have a dramatic influence on the results of a study. Careful patient stratification or direct correlations between structural anomalies and behavioural phenotypes represent important experimental approaches for future studies. As an example of the latter approach, a recent study reported reduced grey matter volume in lobule VII and CrusI/II in ASD subjects, and demonstrated a correlation between these changes and the severity of a number of behavioural and cognitive deficits, which included impaired social interaction, communication and increased repetitive behaviours (D'Mello et al. 2015).

One of the most consistent neuroanatomical abnormalities observed in the postmortem analysis of brains from individuals with ASD is a substantial decrease in the size and number of Purkinje cells, primarily in the posterolateral neocerebellar and archicerebellar cortices (Fatemi et al. 2012). As Purkinje neurons are the sole output of the cerebellum, these findings may have important functional relevance. Data from 24 postmortem studies showed a striking 79% incidence of significantly decreased Purkinje cell numbers in the cerebellar hemispheres of ASD brains (Amaral et al. 2008). A recent study by Wegiel et al. (2014b) included the first unbiased stereological assessment of Purkinje cell size and morphology in ASD. They describe a reduction of 25% in the total number and a 24% decrease in the density of Purkinje cells in the cerebella of 14 subjects with ASD vs. 14 age‐matched controls (4–60 years old) postmortem. Another study by Wegiel et al. (2014a) has also shown significant reductions in Purkinje cell volume of ~31% in 4‐ to 8‐year‐olds and ~23% in 29‐ to 60‐year‐olds.

Despite the relatively strong experimental evidence of Purkinje hypocellularity in ASD, some caveats still remain. Some studies have returned only a small subset of autistic individuals with defects in Purkinje cells (Whitney et al. 2008), consistent with the genetic heterogeneity of ASD. Cerebellar Purkinje neuron development might also be particularly susceptible to compounds such as valproic acid that might trigger ASD symptoms (Ingram et al. 2000), introducing yet another potential source of variability. It is important to remember that postmortem observations might not be directly or causally linked to ASD. Psychoactive drugs used to treat ASD symptoms might over the long‐term be responsible for some of the changes observed in postmortem studies. Furthermore, some findings in postmortem studies may be because of compensatory structural changes, rather than representing developmental causes of ASD.

These anatomical findings also lead to a critical question: are the Purkinje neuron deficits sufficient to cause ASD? Recent studies in the mouse support a causative role for Purkinje cell deficits in ASD. The inactivation of the tuberous sclerosis gene *Tsc1* specifically within Purkinje cells of the developing cerebellum caused a range of autism‐like behavioural changes in these mice (Tsai et al. 2012). By contrast, a recent study in our laboratory, found that a substantial (> 50%) reduction in Purkinje cell number caused by reduced postnatal cerebellar growth was not sufficient to produce autism‐like behaviours in mice (Whittaker et al. submitted). In this study, Purkinje cells were not targeted directly but their numbers were reduced as a consequence of deleting a gene in cerebellar granule cell lineage, which is responsible for driving postnatal cerebellar growth. Taken together, these findings suggest that the developmental origin and underlying causes of Purkinje neuron abnormalities may be critical determinants of their behavioural consequences.

The neurobiological basis by which cerebellar dysfunction might give rise to ASD is still heavily debated (Bauman & Kemper, 2005; Basson & Wingate, 2013). Evidence from primates support a model whereby the cerebellum modulates the activity of cortical areas implicated in executive and other functions disrupted in ASD, through cerebro‐cerebellar connections (Strick et al. 2009; D'Mello et al. 2015). Wang et al. (2014) have also suggested that specific regions of the cerebellum influence remote neocortical areas relating to social interaction, and claim that a sensitive period of disruption of these mechanisms of communication between the cerebellum and cortex could account for some of the behavioural phenotypes associated with ASD.

The involvement of the cerebellum in cognition is now firmly established. Functional MRI (fMRI) studies have identified cerebellar activation in a range of cognitive tasks such as language, visual, spatial, executive and working memory (Stoodley, 2012). A meta‐analysis of 350 fMRI studies showed little evidence for the cerebellum being involved in social thought that requires little or no abstraction (Van Overwalle et al. 2014). Their study does, however, suggest an important, but perhaps not essential, role for the cerebellum in social cognition that requires abstraction away from the current social interaction, i.e. cognition that includes episodic autobiographic memory or hypotheticals. However, the authors remark that their analysis was somewhat limited by the lack of available fMRI data for the cerebellum. For a comprehensive review of important discoveries regarding the cerebellum and cognitive function see Becker & Stoodley (2013).

Overall, it is clear that there is considerable variation between studies focused on the role of the cerebellum in ASD, with many seemingly conflicting results being published. It is encouraging, on the other hand, to see studies observing reproducible links between abnormalities in the structure of the cerebellum and distinct behavioural phenotypes (D'Mello et al. 2015). Animal studies, particularly in the mouse, are continuing to provide valuable insight into how specific cerebellar defects may lead to ASD‐like behaviours. The ability to functionally inactivate defined cerebellar regions and circuits and assess the behavioural consequences is likely to yield important insights in the near future.

### Amygdala

The amygdala is an almond‐shaped group of nuclei found deep in the anterior medial temporal lobe (Sah et al. 2003) and has functions in emotional processing, including fear, pleasure and aggression. Impairments in social interaction, prediction of reward, emotional memory, and facial and emotional recognition in ASD may therefore be indicative of malfunction of the amygdala and associated structures (Davis & Whalen, 2001). A number of studies in the past decade have explored the role of aberrant growth patterns of the amygdala in children and adolescents with ASD (reviewed in Gadad et al. 2013).

Kemper & Bauman (1993) were among the first to observe abnormalities in the amygdala of autistic individuals, finding increased cell densities and decreased neuronal cell sizes. However, a small sample size of only six cases, four of which with a comorbid seizure condition, could have contributed to the anatomical abnormalities observed.

The amygdala later returned to the forefront of ASD research, with a number of studies suggesting a role in the neuropathology of ASD. For instance, Schumann & Amaral (2006) carried out stereological analyses of nine adult male postmortem brains of patients with ASD and found no difference in the total volume of the amygdala or cell size, but they did report reductions in the number of neurons in the amygdalae of autistic individuals compared with age‐matched controls.

Later studies by Schumann reported changes in the relative size of the amygdala specifically in children. An analysis of MRI data from 89 children (1–5 years of age) found that the sizes of the right and left amygdala, relative to total brain volume, were increased in toddlers with a confirmed diagnosis of ASD compared with controls (Schumann et al. 2009). Intriguingly, it was also reported that the degree of amygdala enlargement in toddlers was positively correlated with the extent of their social interaction and communication deficits at 5 years of age.

A number of other studies have shown incidences of increased amygdalar volume in early life (< 5 years of age) in autistic individuals (Munson et al. 2006; Mosconi et al. 2009). Schumann et al. (2004) previously found no evidence of amygdala enlargement in adolescents with ASD, suggesting that amygdalar size and cell number alterations in ASD are age‐dependent. Therefore, the current consensus regarding amygdalar volume and ASD seems to be that a precocious enlargement of the amygdala in early postnatal life eventually slows down and even reverses, such that the amygdala in adults with ASD may have fewer neurons than controls. However, it is clear that larger, well‐controlled longitudinal studies will be necessary to support this hypothesis.

As with other brain regions, understanding the contribution of amygdalar dysfunction to ASD symptoms is helped immeasurably by studies in animal models. Lesion studies in primates have suggested a role for the amygdala in increased anxiety associated with ASD (Amaral et al. 2003) but did not find any clear link with the deficits seen in social behaviours. However, a more recent study by Hong et al. (2014) used pharmacological and optogenetic cell type‐specific functional manipulations to show that distinct neuronal subpopulations in the medial amygdala were important for aspects of social and asocial behaviours. A GABAergic subpopulation of neurons in the posterior dorsal medial amygdala was found to promote behaviours related to aggression, mating and social grooming, whereas a glutamatergic subpopulation in the same region promoted asocial behaviours such as self‐grooming. These two populations of neurons were also seen to act antagonistically, with each inhibiting the behaviours regulated by the other. The authors remark that their observations seem strikingly reflected by the deficits of social behaviour in humans with ASD, and that they fit well with the excitatory : inhibitory imbalance theory of autism (Rubenstein & Merzenich 2003).

### Frontal cortex

The frontal cortex controls many of the executive functions of the brain, including higher‐order cognitive processes such as decision‐making, planning, working memory, emotions, social behaviour, learning and communication. Due to the impairments in social interaction and emotion seen in ASD, the frontal cortex has also been a region of the brain that has received considerable attention in recent years. The main observations in ASD include abnormal cortical growth patterns, abnormalities in cortical thickness and disorganisation of neurons across the cortical layers and their connections to other regions of the brain.

The results from a number of longitudinal studies of volumetric brain changes in ASD have been reported in recent years. These studies have allowed for much more comprehensive views of the changes in brain growth that occur in children and persist in adults with ASD, and could one day be used to aid current clinical diagnosis.

The results from the first longitudinal study of cerebral cortical development in toddlers diagnosed with ASD were reported by Schumann et al. (2010). Previous MRI‐based studies had suggested that the brains of children with ASD underwent abnormal growth patterns with precocious overgrowth of the frontal cortex (Carper & Courchesne, 2005). Schumann et al. therefore examined MRI scans from 41 toddlers with a confirmed ASD diagnosis and 44 controls with typical brain development, at a number of time points. A significant 7% enlargement in the total size of the cerebrum was seen in autistic toddlers compared with the controls up to 2.5 years of age, with a 10 and 5% increase in total white and grey matter, respectively. This clearly supported the suggestions of previous studies, namely that there are abnormal patterns of cortical growth in toddlers with ASD and that the overgrowth begins before 2 years of age. It further highlighted the need to include younger children in such studies to more accurately define the developmental disruptions associated with ASD.

The largest MRI study to date comparing ASD and neurotypical brain size was published by Courchesne et al. (2011a). This study examined the brains of individuals between 1 and 50 years of age and, akin to previous studies, reported evidence of early frontal cortex overgrowth followed by a marked reduction in brain size in ASD cases (Courchesne et al. 2011a). A postmortem study by Courchesne et al. (2011b) in the same year found complementary results, namely an increase in neurons and total weight in the dorsolateral and medial prefrontal cortices in children with ASD compared with the controls. This study, as with most postmortem studies, was limited by a small sample size of only seven brains of children with ASD and six controls, making the results perhaps even more striking.

More recently, Zielinski et al. (2014) carried out another large longitudinal study looking at changes in cortical thickness. It encompassed various time points in postnatal development in 97 males with ASD (3–36 years old) and 60 males with typical brain development (4–39 years old). Their results and observations were consistent with previous studies, showing increased cortical thickness in early childhood followed by a decline in growth and arrest of development in later childhood that persisted into adolescence. They reported normalisation of cortical thickness in mid‐ to late childhood (8–18 years), although they did highlight the possibility that this might not be indicative of any wider structural or functional normalisation.

To try and resolve some opposing views on whether cortical surface area or thickness is more significantly altered in young children with ASD, Ohta et al. (2016) compared cortical thickness and surface area in 112 boys of 3 years of age with ASD with 50 typically developing age‐matched controls. They provided evidence that the observed increases in cortical grey matter volume in boys with ASD were almost solely down to increases in cortical surface area and not thickness. This finding suggests that the early overgrowth of the cortex is more likely associated with an increase in the cortical surface area, rather than an increase in cortical thickness. However, this point remains contentious, as other MRI studies have remarked that cortical thickness is still proven to be the most reliable observation between ASD and neurotypical brains. For instance, a fairly recent study from the UK used multiple parameters, including cortical thickness, volumetric features, and geometric features, to distinguish brain anatomy of autistic adults from neurotypical adults. Although much improved by taking all features into account, cortical thickness by itself was still considered to provide the most accurate classification (Ecker et al. 2010).

Currently, the largest ongoing single site longitudinal study of MRI data from young children with Autism Spectrum Disorders is the Autism phenome project (http://www.ucdmc.ucdavis.edu/mindinstitute/research/app/), which has collected data from over 300 families to date. MRI scans are currently being collected in three categories; families with girls between 2 and 3½ years old, families with boys between 2 and 3½ years old and children between 9 and 12 years old. The results from this study should prove invaluable in defining the dynamic neuroanatomical changes that appear to characterise the complex trajectory of brain development in individuals with ASD.

As we have seen in the amygdala, optogenetic dissection in model systems can be a valuable tool for understanding of the role of distinct brain regions and cell populations in specific behaviours. Yizhar et al. (2011) used optogenetic techniques directly to perturb the excitatory : inhibitory balance in the neocortex. Under certain conditions it was found that elevation of cellular excitatory : inhibitory balance in the medial prefrontal cortex of the mouse elicited specific impairments in social behaviours. They also managed to rescue this phenotype partially by increasing inhibitory tone, further providing support for the theory that increased excitatory : inhibitory balance can lead to the behavioural defects seen in ASD.

In conclusion, convincing evidence is mounting to support the view that the trajectory of brain growth is altered in ASD. Both the amygdala and frontal cortex appear to overgrow during the first few postnatal years, followed by a normalisation, or even decrease, in volume or cellularity, compared with neurotypical controls. Although cerebellar hypoplasia and Purkinje cell hypocellularity have been reported, the developmental trajectory of cerebellar development in individuals with ASD has not, to our knowledge, been investigated. Large‐scale, longitudinal studies will help determine the scale and incidence of anatomical differences during cerebellar, amygdalar and cortical development. Consistent with the heterogeneous nature of ASD, several other brain regions may be affected in ASD. Some regions that are of particular interest include the deep cerebellar nuclei, limbic system and other brain stem nuclei. For a comprehensive review, see Blatt (2012). Clearly, a great deal still needs to be learned about the normal development and function of the different brain regions implicated in ASD before the consequences of abnormal development can be fully appreciated.

Having focused primarily on the neuroanatomy ASD at the mesoscale, we move on to discuss microscopic findings which may point towards the cellular and developmental basis of these neuroanatomical abnormalities and are likely to underlie some of the pathophysiological alterations in brain circuitry associated with ASD.

## Cortical microstructure abnormalities

### Alterations in the columnar structure of the neocortex

Neuronal cell bodies in the neocortex are ‘stacked’ on top of each other to form so‐called minicolumns. Mountcastle first described minicolumns and provided electrophysiological evidence that these anatomically defined modules represent elemental units of information processing (Mountcastle, 1957, 1997). However, the exact function of cortical minicolumns is still a matter of debate (Bugbee & Goldman‐Rakic, 1983; Opris & Casanova, 2014). Hofman (2001) proposed that minicolumns allow a larger number of neurons to be connected by fewer axons, for increased neuronal connectivity, while allowing short‐range, modulatory connections, especially important for integration during complex tasks requiring executive function, an aspect typically affected in neurodevelopmental disorders like ASD.

Casanova et al. (2002, 2003, 2006) have examined the microstructure of layer III in the dorsolateral prefrontal cortex and Brodman's area 9 (BA9) in 14 ASD postmortem samples and reported a reduced spacing between minicolumns. By contrast, McKavanagh et al. (2015) reported wider minicolumns in the primary auditory, auditory association, orbital frontal and parietal cortex of ASD brains, particularly in younger individuals, again supporting the notion of an abnormal developmental trajectory in ASD. Intriguingly, McKavanagh et al. re‐analysed the data from Casanova et al. (2006) taking into account age and found exactly the same trend of increased minicolumn width in ASD patients at younger ages. Taken together, these findings are consistent with an altered trajectory of brain growth in ASD. Nevertheless, sample sizes remain small and assessment of minicolumn width in more brain samples is needed. Interestingly, Casanova et al. (2006) report in one of their studies that pyramidal cell somata were smaller in their ASD brain samples. This is in contrast to the expectation of ASD subtypes caused by mutations in *PTEN* and resulting hyperactivation of the mTOR signalling pathway, where somatic hypertrophy is expected (Zhou & Parada, 2012). This finding again underscores the importance of future attempts to stratify patient samples based on the underlying genetics.

Despite these uncertainties, these findings raise important fundamental questions. What are the mechanisms that establish minicolumnar organisation during brain development and how exactly is the spacing between them regulated? What exactly are the functional consequences of altered minicolumnar organisation? Are there key differences in minicolumn organisation in different organisms, and what is the evolutionary significance of this? Even more fundamentally, can it reasonably be assumed that minicolumns represent an intrinsic, uniform modular organisation that can be compared between different individuals? Recent high‐resolution construction of macro‐columnar organisation in the rat somatosensory cortex suggests that the number of neurons within these larger, functional aggregates varies substantially between different individuals and is correlated with sensory input (Meyer et al. 2013), suggesting that much more is to be learnt about the different levels of columnar organisation of neurons in the cortex.

**Figure 1:**
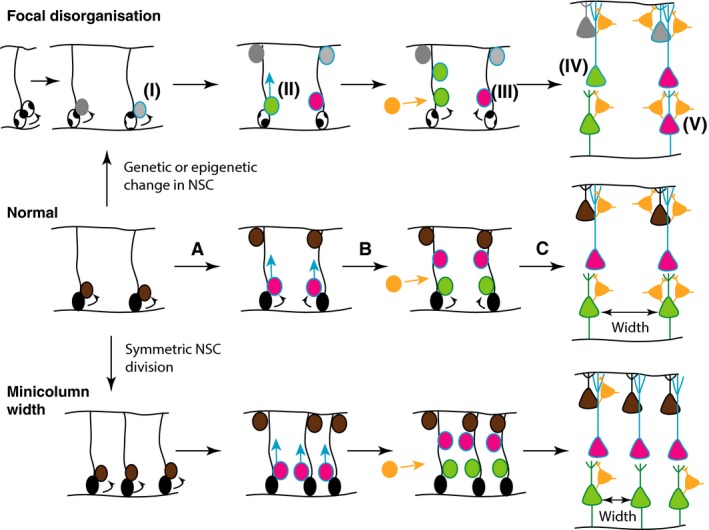
Hypothesis of potential mechanisms underlying focal disorganisation and altered minicolumn distribution in ASD cortex. Normal: During normal cortical development (middle panel), radial glial NSCs (black) located at the ventricular zone, with radial processes extending to the pial surface of the neural epithelium, can undergo symmetrical (to produce more radial glial NSCs) or asymmetric divisions to maintain the NSC pool and generate committed neural progenitors (brown) (A). The latter migrate towards the pial surface on the radial scaffold, where they differentiate into projection neurons. This process continues with different classes of neurons (pink, green), being produced in successive waves over developmental time (B), so that different neuronal types are grouped together in different cortical layers (C). GABAergic interneurons (orange) migrate into the neuroepithelium (B), and integrate into the circuitry (C). At the end of this process, projection neurons are organised in radial minicolumns, a reminder of their developmental origin. Focal disorganisation: We hypothesise that focal disruptions may be the result of early genetic or epigenetic changes in NSCs (i), such that the resulting neuronal progenitors have defects in differentiation (ii) or migration (iii) such that they adopt the wrong fate (iv), or end up in an in appropriate position in the brain (v). The end result is a cortex with apparent disorganisation of neuronal cell types and perhaps even distorted cell densities. Minicolumn width: We hypothesise that alterations in symmetric NSC divisions such that the early density of radial scaffolds is increased, will result in increased density of minicolumns. Alternatively, the migration and integration of GABAergic interneurons and other independent mechanisms might affect the spacing of minicolumns.

The basic modular organisation is most likely the result of the developmental process of neurogenesis, when newly born projection neurons migrate along radial glial fibres from the ventricular layers towards the pial surface (Fig. 1). The final spacing of the minicolumns is therefore most likely a function of the density of these glial scaffolds during development (Fig. 1). However, the exact details of how this process is regulated spatially and molecularly remains largely unexplored. It is tempting to speculate that the Notch signalling pathway that controls radial glial neural stem cell (NSC) and neural progenitor fates might be involved. These Notch‐regulated processes might very well be sensitive to dysregulated signalling pathways in ASD, which may include the WNT and Reelin pathways (Cotter et al. 1999; Lakoma et al. 2011).

### Focal disorganisation in the neocortex

A recent study by Stoner et al. (2014) examined postmortem prefrontal, temporal and occipital neocortical tissue from 11 children with ASD and 11 typically developing controls between 2 and 15 years of age. *In situ* hybridisation studies on tissue sections revealed patches of reduced or abnormal gene expression that were between 5 and 7 mm in length in 10/11 patients and 1/11 controls. The nature of these alterations varied between different samples, suggestive of a marked heterogeneity. These changes are likely due to defects in neuronal proliferation, differentiation, migration and/or survival, ultimately leading to mis‐positioning of neurons within the wrong layers. Mechanistic studies in appropriate model systems will be required to identify the exact mechanisms responsible. In general, glial‐specific marker expression was normal, ruling out an overt change in glial cell identity.

## Alterations in neuronal subtype distribution

We have learnt a great deal over the last few years about subtype diversification during cortical development. Perhaps most fundamental is the fact that GABAergic interneurons originate from extracortical subpallial progenitor zones (Anderson et al. 1997), in contrast to the excitatory projections neurons that are born within the cortical plate. Dysregulated neurogenesis in one but not the other anatomically distinct neurogenic region may result in a paucity of one neuronal subtype compared with the other, such that the excitatory : inhibitory (E : I) balance is disturbed. Altered excitatory : inhibitory balance is one of the central hypotheses of ASD aetiology (Rubenstein & Merzenich, 2003). A recent report suggested that experimentally increasing the number of excitatory neurons in layers II/III of the neocortex in the mouse is sufficient to cause autism‐like behaviours (Fang et al. 2014), providing compelling support for the E : I imbalance model of ASD. Similarly, altering the balance of parvalbumin : somatostatin‐positive interneurons also caused autism‐like behaviours, consistent with the notion that the ratios of both excitatory and inhibitory neuronal classes have to be tightly regulated for normal function (Vogt et al. 2015).

## Connectivity

The alterations in specific brain regions discussed so far, which may include changes in neuronal density, number, organisation, and composition, are likely to alter the local connectivity in specific regions of the brain. Two major hypotheses have been proposed. One relates to changes in the excitatory : inhibitory balance caused by changes in the composition of different neuronal cell types or activities in the brain (see above). The second proposes over‐connectivity in specific brain areas, an hypothesis based on studies in adolescents with ASD (Keown et al. 2013).

Another aspect of brain anatomy relates to long‐range connectivity, the connection of different brain regions by major axon tracts. Substantial evidence, primarily based on functional MRI and DTI measurements, supports the idea that long‐range connectivity is altered in ASD (reviewed in Vissers et al. 2012). For example, defects in cerebellar‐thalamic‐frontal cortical connectivity may underlie some cases of ASD (reviewed in Basson & Wingate, 2013). Solso et al. (2016) recently reported DTI measurements on 94 ASD and typically developing toddlers (aged 1–4 years old; Solso et al. 2016). They found increased volumes of frontal tracts in toddlers with ASD in earlier years, with a subsequent age‐related deceleration of growth over time, seemingly reflective of the trajectory of volumetric changes seen in MRI studies of the cortex in ASD (Carper & Courchesne, 2005; Schumann et al. 2010; Courchesne et al. 2011a,b; Zielinski et al. 2014).

The exact developmental reasons for these alterations are not known but it is intriguing to note that many ASD‐associated genes have established roles in axon pathfinding (Sbacchi et al. 2010). Recent technical advances such as CLARITY will be instrumental in revealing some of these long‐range connections and specific defects in brains from genetically modified mice (Chung et al. 2013).

Another largely unexplored possibility is that changes in differential growth and maturation of specific brain regions might directly impact on the development and function of other areas to which they are connected. A recent study by the DiCicco‐Bloom laboratory provided evidence that developmental abnormalities in the hindbrain locus coeruleus of Engrailed 2 (*En2*) mutant mice was associated with abnormalities in hippocampal development (Genestine et al. 2015). They hypothesise that defective monoamine transfer to the forebrain from locus coeruleus‐derived fibre tracts was responsible for these defects, as a norepinephrine agonist could reverse some of these defects. Intriguingly, monoamines can also modulate minicolumnar circuits (see for example Casanova, 2007), providing yet another possible mechanism of how the development of structures in the hindbrain might modulate cortical development and function. It is pertinent to note here that correlations between cerebellar and frontal cortex volume have been reported, suggesting that these regions are not merely connected to modulate each other's function, but possibly also each other's growth trajectories (Carper & Courchesne, 2000). The widely used conditional gene deletion strategies where genes can be specifically disrupted in defined anatomical locations during brain development (e.g. the hindbrain) and investigating the consequences of these on the development of a distant, but interconnected region (e.g. the cortex) should be particularly well‐suited to address these questions.

## Changes in regional identity

With the recent advances in next generation sequencing approaches, the epigenetic and transcriptomic profiling of different brain regions or even individual cells has become possible. A recent study has compared the transcriptome of specific brain regions in ASD and control postmortem brain tissue (Voineagu, 2012). In addition to identifying interesting new functional modules of gene expression changes in ASD brains, an intriguing finding from these studies was that the distinct gene expression profiles that can typically distinguish different brain regions, e.g. the frontal and temporal cortex from each other, were much less dissimilar in ASD brains (Voineagu, 2012). This implies that the factors that determine the molecular (and by inference functional) identity of different brain regions are attenuated in ASD. If these findings are validated in larger sample sizes and found to accurately reflect the situation in specific ASD subtypes, this would imply that alterations in regional identity, perhaps without overt differences in size, might be associated with the ASD phenotype.

Chow et al. (2012) also identified abnormal gene expression patterns in ASD using whole‐genome analysis of mRNA levels and copy number variations in postmortem brain samples from autistic and neurotypical individuals (Chow et al. 2012). A number of genes involved in brain patterning were downregulated in younger ASD brains. They also observed dysregulation of genes involved in cellular differentiation in adult ASD brains, which could contribute to the reported reduction in ASD brain growth over time (Courchesne et al. 2011a). It is promising that these data reflect the age‐dependent changes in cortical growth identified in other studies, and shows the continuing progress towards finding mechanistic explanations for these observations. It should be noted that these gene expression studies also identified expression differences in genes involved in the DNA damage response, proliferation, apoptosis and survival that could contribute to the cortical growth and focal dysplasia phenotypes described earlier.

## Understanding the fundamental mechanisms regulating brain development

Summarising our discussion thus far, many cases of ASD are associated with an expanded size of the frontal cortex during the first few years of life. Other reports suggest differences in size of several other brain regions and even epigenetic alterations suggestive of altered identity. On a cellular scale, cortical dysgenesis, which includes changes in cortical thickness, neuronal and minicolumn density, and focal disorganisation have been reported in ASD. These findings of abnormal anatomy have stimulated renewed interest in some of the fundamental processes that regulate brain development. In this section, we will review these and define specific testable hypotheses of the dysregulated mechanisms that might underlie specific autism‐associated changes.

We should acknowledge that much of what we have learnt about mammalian brain development has been gleaned from studies in the mouse. The ability to generate mouse mutants with loss, and sometimes gain of function alleles, often in a region‐ or cell type‐specific manner and with tight temporal control has been a powerful tool to manipulate specific processes and dissect the underlying molecular and cellular mechanisms. However, this model system also has its limitations. Substantial intrinsic differences exist between mouse and human cortical development, most notably in the temporal aspect (Espuny‐Camacho et al. 2013). Thus, it might not always be possible in the mouse to model accurately the abnormal brain growth trajectories observed in humans over developmental time.

## Mechanisms regulating region‐specific brain size

### Patterning and regionalisation

From induction of the neural plate during early embryonic development, a combination of intrinsic (transcription factor, non‐coding RNAs and other epigenetic regulators) and extrinsic (secreted and membrane‐bound signalling molecules) factors induces gene expression patterns that define different brain regions. From an initial broad establishment of anterior‐posterior and dorsal‐ventral axes, subsequent secondary organisers pattern the developing neural tube further (Kiecker & Lumsden, 2012). The best‐characterised transcription factors include homeobox transcription such as OTX2 that defines anterior neural tube and GBX2 that defines, in a complementary pattern, posterior neural tube. The fibroblast growth factors (FGFs), in particular FGF8 and FGF17, are critical for the establishment of the frontal cortex (Cholfin & Rubenstein, 2008; Assimacopoulos et al. 2012). The same factors are involved in regionalisation of the cerebellum, where reduced FGF8 and FGF17 signalling underlies cerebellar vermis hypoplasia in some instances (Basson et al. 2008; Yu et al. 2011). A reduction in dorsomedial prefrontal cortex size in *Fgf17* ^*–/–*^ mice is associated with reduced social behaviours (Cholfin & Rubenstein, 2007; Scearce‐Levie et al. 2008). This latter study shows that studies of frontal cortex patterning in genetically defined mouse mutants can be very powerful to link specific anatomical changes to behavioural abnormalities.

### Neural stem and progenitor cell (NSC) expansion

During early brain development, NSCs in the neuroepithelium divide symmetrically to increase the size of the brain and the NSC pool. The loss of certain growth factors appears to affect primarily the expansion and/or survival of NSCs in specific regions. For example, loss of FGF2 dramatically affects the size of the cortex, whereas basal structures appear normal. Pyramidal neuron number is disproportionally reduced in the frontal compared with the parietal cortex of *Fgf2*‐deficient mice (Raballo et al. 2000). *Fgf10* mutant mice represent another intriguing example. FGF10 is required for the transition of symmetrically dividing neuroepithelial cells into radial glial‐like NSCs. A delay in this transition in *Fgf10* ^*–/–*^ mice results in an expansion of the NSC pool, particularly in the rostral cortex, such that the frontal cortex is larger and thicker (Sahara & O'Leary, 2009). Similar region‐specific effects on NSC expansion during late embryonic and early postnatal development might underlie the differences in regional size and overgrowth reported in ASD brains.

Genetically defined mouse models have clearly been instrumental in elucidating some of the fundamental mechanisms regulating neurogenesis and brain size. Despite these interesting examples, the actual genes and pathways driving the expansion of frontal cortex and brain size in ASD have not been identified. Genotype : phenotype correlation data from patients that can then be translated to appropriate model systems are necessary to make substantial progress in this field.

An alternative to modelling specific features of brain development in the mouse, involves the generation of induced pluripotent stem cell (iPSC)‐derived human brain organoids. A recent study produced such organoids from patients with ASD and macrocephaly and compared these with organoids derived from unaffected family members (Mariani et al. 2015). These organoids were found to represent telencephalic neuronal progenitors at mid‐fetal stages of development. Perhaps not that surprisingly, given that they were generated from patients with macrocephaly, but certainly encouraging for this approach, these progenitors were found to have a shortened cell cycle and an increased tendency to generate neurons. They also showed an increased tendency to generate inhibitory neurons. Transcriptomic analyses of the organoids revealed upregulated FOXG1 expression in the ASD cases that was at least partly responsible for the increased expansion of early GABAergic neural progenitors (Mariani et al. 2015). It is important to note that the E:I imbalance proposed as pathological mechanism by this study is the opposite to the mouse study by Fang et al. (2014) discussed earlier. Thus, if the *in vitro* organoid model system does indeed reflect the real situation *in vivo*, one would conclude that excitatory : inhibitory balance can be disturbed in either direction in ASD, perhaps dependent upon the underlying genetic and environmental causes.

### Mechanisms involved in transient brain overgrowth

Early overgrowth of the brain, most notably involving the prefrontal cortex, is a common feature of ASD (see above). Recent longitudinal studies have confirmed earlier work that has associated increased brain size (and macrocephaly) with ASD. It should be noted that not all cases of ASD are associated with increased brain growth and macrocephaly. Many patients have a brain and head size within normal range and, indeed, microcephaly is also over‐represented within the ASD population (van Bon et al. 2015).

Clearly, the same mechanisms outlined in the previous section are likely to play a role here. It is likely that early regionalisation, NSC expansion, and changes in modes of division and differentiation underlie these effects. A recent analysis of brain growth in *Pten* ^*+/−*^ mouse mutants confirmed increased brain size in these mice from the day of birth through to adulthood (Chen et al. 2015b), similar to findings in humans with autism‐associated *PTEN* mutations (Butler et al. 2005). Intriguingly, whereas increased brain size during early life was caused by increased numbers of neurons in *Pten* ^*+/−*^ mice, consistent with an increased generation of neurons by NSC proliferation, the neuron : glial ratio was significantly reduced in adult brains. Apoptosis was increased at P4, suggesting that excess neurons may be eliminated by apoptosis postnatally (Chen et al. 2015b). These findings are consistent with a mechanism whereby abnormal levels of NSC proliferation during late embryogenesis results in increased brain growth during early postnatal life and macrocephaly. Clearly, homeostatic mechanisms exist that can lead to the elimination of excess neurons and connections later on in life, leading to a reduction in the relative numbers of neurons and might even in some instances be associated with a substantial reduction in brain growth (Schumann et al. 2010). Intriguingly, the same pathways associated with transient brain overgrowth, also regulate synaptic spine pruning during adolescence (Tang et al. 2014).

## Cellular mechanisms responsible for microanatomical abnormalities

On a cellular scale, cortical dysgenesis, which include changes in cortical thickness, neuronal and minicolumn density, and focal disorganisation have now been reported in ASD (see above). These findings clearly implicate abnormalities during the establishment of the normal neocortical microarchitecture in ASD pathogenesis. However, many questions still remain. The developmental process(es) disrupted and ultimately responsible for the altered neuroanatomy are not known. Furthermore, the exact causal relationship between these neuroanatomical alterations and behavioural features of ASD has not been addressed. Several mechanisms can be envisaged and have indeed been proposed. Ultimately, testing these hypotheses will rely heavily on evidence from mouse models.

### Altered neural stem cell self‐renewal and progenitor expansion

Alterations in neurogenesis may affect minicolumn distribution and underlie the formation of abnormal patches observed in ASD postmortem brains (Fig. 1). An increased density of NSCs and therefore radial glia should give rise to minicolumns that are more densely packed. Alternatively, a paucity of GABAergic interneurons that populate the space in‐between the minicolumns might underlie these changes. A careful anatomical analysis of the consequences of experimentally manipulating NSC density and interneuron numbers will be necessary to determine whether either of these is sufficient to change the intercolumnar width in the mouse cortex. As far as focal anomalies go, one would need to propose a fairly dramatic change in an early NSC that expands and gives rise to a small (5–7 mm) patch of abnormal neuronal progenitor cells (Fig. 1). In that sense, there might be significant clinical overlap between these ASD cases and focal dysplasias. It is intriguing to note that mutations in the PTEN‐mTOR pathway have been linked to cortical dysplasia and epilepsy, providing a potential mechanistic overlap with ASD and perhaps also suggesting intriguing hypotheses on the basis of epileptic comorbidity with ASD (Lim et al. 2015).

### Migration defects

Radial migration abnormalities might also underlie the recently described abnormal microfoci in autism neocortices (Stoner et al. 2014). However, no overt glial abnormalities were observed in the majority of brains assayed in this study by Stoner et al., suggesting that disorganisation of radial glia is unlikely to be the primary cause of these neuroanatomical changes. Many autism‐associated genes are known to have functions in neuronal migration, e.g. *RELN* and focal expression of hyperactive mTOR are sufficient to disrupt neuronal migration (Lim et al. 2015), providing indirect support for migration defects as a pathogenic mechanism.

## Conclusions

Given the heterogeneity of ASD, based on behavioural phenotype and genetic underpinnings, it is reasonable to assume that it is unlikely that one single neuroanatomical alteration or developmental abnormality will be found to underlie the majority of ASD pathology. Despite this, it is intriguing that genes identified in ASD tend to cluster functionally within a few defined functional groups. These include genes and pathways associated with synaptogenesis, neuronal migration and axon guidance, chromatin remodelling and developmental signalling pathways such as WNT (Geschwind, 2011; Krumm et al. 2014; Chen et al. 2015a). This gives us hope that, rather than dealing with as many conditions as there are genes associated with ASD, which now run into the hundreds, we might be dealing with perhaps fewer than 10 ASD subgroups that would be definable by genetic and underlying pathological mechanism(s).

## Future prospects

The concerted efforts to identify the functionally‐relevant neuroanatomical changes in ASD, will not only provide important insights into how relatively subtle changes during brain development can result in autistic behaviours, but will also inform us of the normal developmental programmes that control brain development and the functional importance of specific neuroanatomical features of the brain. These studies will identify the mechanisms that control and restrain brain growth over developmental time. Important questions posed in this review will be answered. For example, are the neuroanatomical features observed in ASD, such as prefrontal cortex overgrowth, cerebellar Purkinje neuron hypocellularity or focal dysplasias in the cortex, sufficient to cause ASD? The ability to define ASD subtypes based on genetic aetiology, careful anatomical analyses of these different genetic subgroups, and genetic studies in model organisms and systems to understand the developmental underpinnings and behavioural consequences of specific genetic changes will be necessary to provide satisfactory answers to these questions.
